# BBBomics-Human Blood Brain Barrier Transcriptomics Hub

**DOI:** 10.3389/fnins.2016.00071

**Published:** 2016-03-01

**Authors:** Krishna R. Kalari, Kevin J. Thompson, Asha A. Nair, Xiaojia Tang, Matthew A. Bockol, Navya Jhawar, Suresh K. Swaminathan, Val J. Lowe, Karunya K. Kandimalla

**Affiliations:** ^1^Division of Biostatistics and Bioinformatics, Department of Health Sciences Research, Mayo ClinicRochester, MN, USA; ^2^Department of Pharmaceutics and Brain Barriers Research Center, University of MinnesotaMinneapolis, MN, USA; ^3^Department of Radiology, Mayo ClinicRochester, MN, USA

**Keywords:** blood brain barrier, transcriptomics, RNA-seq, microRNA-seq, pathways, informatics analysis, BBB database

## Introduction

Blood-brain barrier (BBB) is a monolayer of endothelial cells that line brain capillaries. The BBB protects brain by blocking the entry of harmful substances from blood and shielding the brain from peripheral fluctuations in hormones, fatty acids, and electrolytes. In addition, the BBB effectively clears brain metabolites and serves as a major conduit for the delivery of crucial nutrients and growth factors needed for proper brain function. Owing to these critical responsibilities, any functional and structural impairment of the BBB may result in severe pathophysiological consequences in the brain. BBB dysfunction is implicated in several neurodegenerative disorders including Alzheimer's disease (Carmeliet and De Strooper, 2012), Parkinson's disease (Kortekaas et al., 2005), and cerebrovascular diseases (Yang and Rosenberg, 2011) such as cerebral amyloid angiopathy, stroke, and vascular dementia. Hence, the research community has been actively investigating the cerebrovascular contributions to neurological diseases with major emphasis on the BBB. The success of these efforts is heavily dependent upon the availability of reliable *in vitro* as well as *in vivo* BBB models.

Polarized monolayers of human cerebrovascular endothelial cells (hCMEC/D3) described in the current work serves as one such *in vitro* model that can be easily cultured and manipulated in the lab (Poller et al., 2008; Vu et al., 2009; Weksler et al., 2013). The barrier properties and the expression of several classes of receptors, transporters, and enzymes in hCMEC/D3 cells have been previously investigated (Urich et al., 2012; Lopez-Ramirez et al., 2013, 2014; Bamji-Mirza et al., 2014; Ilina et al., 2015; Naik et al., 2015; Sajja and Cucullo, 2015). Thus, far, the genomic data for hCMEC/D3 cell lines have been generated using array-based approaches (Lopez-Ramirez et al., 2013). However, a comprehensive transcriptomic landscape of hCMEC/D3 cells, which is required for investigating molecular mechanisms using sophisticated computational biology approaches, is not currently available.

Next-generation sequencing technology unveils the full potential of systems biology approaches to resolve cellular and molecular interaction networks that regulate the functional integrity of the BBB. Such a panoramic view of the interaction networks could enable us to isolate key players regulating a physiological process and investigate how they are affected in various diseases. To our knowledge, we are the first group to generate deep RNA sequencing and microRNA sequencing of a human BBB cell line. This data report describes BBBomics hub as a comprehensive portal for BBB transcriptomics data, obtained by sequencing mRNA (mRNA-seq) and microRNA (miRNA-seq) of polarized hCMEC/D3 cell monolayers. This data encompasses coding (gene expression, alternate splice forms, expressed single nucleotide variants -eSNVs) and non-coding (microRNA, LincRNA, circular RNA) counts that are easily accessible through BBBomics hub database. We also superimposed the RNA-seq coding data on 285 Kyoto Encyclopedia of Genes and Genomes (KEGG) pathways, which include canonical, non-canonical, and/or atypical pathways retrievable using BBBomics hub. The data is easily accessible and freely available at http://bioinformaticstools.mayo.edu/bbbomics/.

## Methods

### Cell culture

The immortalized human cerebral microvascular endothelial cell line (hCMEC/D3) was kindly provided by P-O Couraud, Institut Cochin, France. The polarized endothelial monolayers were generated like described previously (Agyare et al., 2013) and the detailed methods are provided in the Supplementary Methods section.

### Illumina TruSeq v2 mRNA and microRNA protocol

RNA libraries for eight replicates of polarized hCMEC/D3 cell monolayers were prepared according to the manufacturer's instructions using TruSeq RNA Sample Prep Kit v2 (Illumina, San Diego, CA). A detailed protocol has been included in the Supplementary Methods.

### RNA-Seq data analysis

Paired-end RNA-sequencing data alignment and processing was performed using the MAP-RSeq—a comprehensive computational workflow developed at the Mayo Clinic to obtain a variety of genomic features from RNA-seq experiment (Kalari et al., 2014). The main goal of the MAP-RSeq pipeline is to obtain multiple genomic features, such as gene expression, exon counts, fusion transcripts from RNA-seq data. On an average, 114 million paired-end reads (51 bp) per sample were processed through MAP-RSeq workflow. MAP-RSeq provides quality control reports and summary statistics of sample reads. Total number of reads, mapped reads, number of reads mapped to the genome, and the numbers of reads mapped to junctions were also obtained for each sample. The RNA-seq mapping statistics for all eight BBB replicates are provided in Supplementary Table 1.

### Gene expression analysis

Gene expression counts were obtained using HT-Seq module http://www-huber.embl.de/users/anders/HTSeq/doc/count.html from MAP-RSeq (Kalari et al., 2014) pipeline for eight hCMEC/D3 replicates. Conditional quantile normalization (CQN) (Hansen et al., 2012) was applied for gene expression counts; normalized data is also available at BBBomics hub.

### Identification of expressed nucleotide variants

Expressed single nucleotide variants (eSNVs) from RNA-seq were called using the eSNV-Detect—a computational method developed by our group (Tang et al., 2014). The eSNVs observed in the eight replicates were summarized and presented with annotations.

### Alternate splicing analysis

The Miso software was used to evaluate alternative splicing among replicates (Katz et al., 2010). Insertion length distributions were pre-calculated from the MAPRSeqV1 pipeline. Ensembl indexes were constructed from the provided Ensembl hg19, build 37 file. Results were compiled using in-house python scripts. Each table also contains the ENSEMBL gene identifiers, the HUGO gene identifier, start and stop positions, and exon model retention (with the ENSEMBL identifiers).

### Circular RNA (CircRNAs) analysis

The circular RNA workflow, Circ-Seq version 1.0, was used to process eight hCMEC/D3 replicates. The unmapped reads obtained from the MAP-RSeq (Kalari et al., 2014) workflow were used as input. Bowtie version 2.1.0 (Langmead and Salzberg, 2012) was used to align reads to the reference genome. Custom python and bash scripts were used to identify and quantitate reads that supported back-splicing events, i.e., RNA transcripts formed from the splicing of 3′ tail to 5′ head. The BLAT software (Kent, 2002) was used to eliminate false candidates that mapped to multiple locations in the genome. The raw read counts were reported per sample. In order to obtain the RefSeq genes that either overlap or neighbor the circular RNA candidates, intersectBed, and closestBed functions were used from the BedTools suite (Quinlan and Hall, 2010).

### Long intergenic non-coding RNA analysis

To identify long intergenic non-coding RNAs (lincRNAs) present in the control samples, the ICQ-lincRNA version 2.0 (lincRNA workflow), was used. The workflow employs the *de novo* transcriptome assembler StringTie (Pertea et al., 2015) version 1.0.3 to assemble and report all transcripts expressed in the samples. After removal of all known RNA transcripts in Gencode (version 19), novel RNA candidates are identified through a set of filters for size selection, expression, repeat masker, and non-protein coding potential prediction using CPAT (Pertea et al., 2015) and iSeeRNA (Sun et al., 2013) to arrive at the final list of potential lincRNA candidates. The raw and normalized read counts were reported per sample. The raw values for each lincRNA were normalized to a million and corrected for the lincRNA length to obtain the normalized reads. The closestBed function from BedTools suite (Quinlan and Hall, 2010) was used to identify both the neighboring RefSeq genes and their distance to the lincRNA. If the lincRNA was found upstream of the gene, the distance was reported with a negative sign. Alternatively, a positive distance was reported if the lincRNA was found downstream of the RefSeq gene. A distance of zero implies overlapped lincRNA, which shares exons with the RefSeq gene.

### MicroRNA analysis

Two replicates of hCMEC/D3 cell monolayers were processed through the microRNA workflow CAP-miRSeq (Sun et al., 2014), version 1.0. The known microRNAs were called using the miRDeep software (An et al., 2013) (version 2.0.0.5), and were annotated using miRBase (version 19) database (Kozomara and Griffiths-Jones, 2011). The raw and normalized read counts were reported per sample. Raw reads were normalized to a million and further computed by dividing each microRNA raw read count by the total number of microRNA reads to arrive at the normalized reads for each sample. TargetScan (Friedman et al., 2009) was used to obtain the computationally predicted gene targets for all microRNAs reported by the CAP-miRSeq workflow.

### Data processing

FASTQ files from RNA and microRNA sequencing were aligned to the human genome build NCBI 37.1 (GRCh37), which corresponds to human genome assembly hg19 in UCSC database (Karolchik et al., 2014).

### Pathway analysis

Pathways with at least 5 sequenced genes were rendered using the R pathview package, version 1.4.2 (Luo and Brouwer, 2013). Expression gradients for the replicates are depicted with respect to the 25th and 75th quantiles of the pathway expression matrix. Pathway gene features were annotated using KEGGREST, version 1.4.1 (Tenenbaum, 2015) and associated with Hugo gene symbols obtained from the Homo.sapiens package, version 1.1.2 (Team, 2015). RNA expression profiles were overlaid using the R package pathview (Luo and Brouwer, 2013) for 285 of the 291 KEGG pathways, where a minimum of 5 annotated RNA genes were observed. Each gene or node in the pathway diagram is represented by the 8 bands, representing CQN normalized values summarized by their expression means (additional details in Supplementary Methods).

### Data or web portal organization and access

The “BBBomics” site is implemented as a single page web service executed via a Linux /Apache HTTPd/Javascript/JQuery/Boostrap/Perl stack. The query interface allows users to search with gene and microRNA IDs and provides links to relevant GeneCards pages and KEGG pathways. This is the first web portal providing a number of transcriptomic features for any BBB cell line. Its applications are versatile and will be beneficial in identifying coding and noncoding transcripts, mutations (eSNVs), and pathway profiles to perform functional studies. Supplementary Methods section of the manuscript consists of instructions of how to query and interpret the data from BBBomics hub. Data used in this study are deposited in the Gene Expression Omnibus web site at GSE76531.

## Results

Genome wide expression, alternate splice forms, expressed single nucleotide variants; long non-coding RNAs, circular RNAs, and pathway regulation data were generated using paired-end RNA-seq of polarized hCMEC/D3 monolayers. These replicates are expected to provide a time independent and unbiased view of the gene expression, along with the expression of single nucleotide variants, alternate splice forms, long non-coding RNAs, and circular RNAs. Isolation of RNA and miRNA as well as library preparations were performed at the Mayo Clinic sequencing core as indicated in Methods. The processing of RNA-sequencing data was performed using MAP-RSeq (Kalari et al., 2014) and miRNA-seq data was performed using CAP-miRSeq (Sun et al., 2014). All count data obtained for mRNA-seq and miRNA-seq were normalized and summarized as described in the Methods Section.

### Coding RNAs

#### Gene expression

The RNA-seq data with 50 bp paired-end reads consisted of eight biological replicates with a total of 913,653,962 (approximately one billion) sequences for one BBB cell line. The statistics of total sequence reads; mapped reads to genome; mapped reads to junction; and unmapped reads for the eight replicates are summarized in Supplementary Table 1. On an average, each sample has 114 million reads; of which, ~82% of the reads map to genome and 14% of the reads map to exon junctions. Due to the depth of the data generated on this cell line, the RNA gene expression, splice forms, and mutations were investigated and identified reliably. After removing the low expressed genes, there are 13,962 genes that are expressed (median raw gene expression count >32). Both normalized and raw gene counts with annotations can be obtained using the BBBomics hub.

#### Alternate splice forms

Splice form results from compare-miso module (Miso software) were compiled using in-house python scripts. For eight replicate BBB samples, two result tables were generated [representing isoforms raw reads and the calculated PSI (“Percentage Spliced In”)] for each transcript observed in BBBomics hub. For each gene analyzed, we obtained the top isoform expressed in the samples, from the PSI value supported by the read counts; the biological variation across the samples; as well as the distribution of isoforms. Each gene in BBBomics hub contains the ENSEMBL gene identifiers, the HUGO gene identifier, start and stop positions, and exon model retention (with the ENSEMBL identifiers).

#### Single nucleotide variants

**Figure 1:**
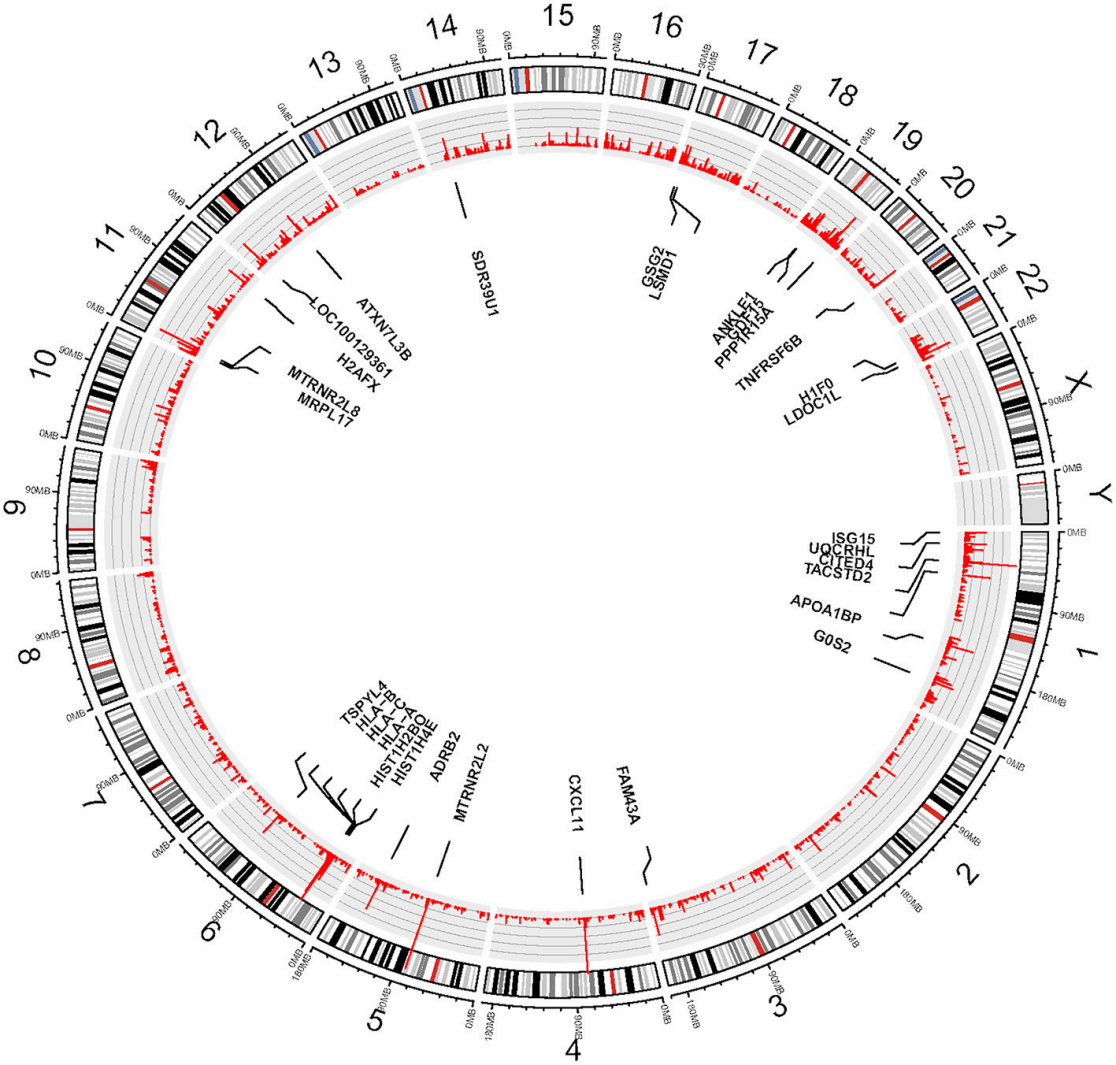
**Circos plot—View of expressed SNVs identified in hCMEC/D3 cell line by chromosomes**. The frequency of the bar indicates the number of times the eSNVs were observed in a sample. The longer the red bar in the inner circle the more frequent the variant was observed in 8 BBB replicates. There are 30 genes identified after normalizing by gene length and numbers of mutations, the gene names are shown in the diagram.

We used eSNV-Detect to identify the expressed SNVs in hCMEC/D3 cell lines. We found 36,057 eSNVs uniquely expressed in the coding region and UTR for the eight replicates. Moreover, the genomic position, confidence level, annotation as well as frequency for all replicates were reported. Among the 36,057 eSNVs, 12,388 (34.5%) were found in the coding region, while 23,668 (65.6%) were found in the UTR. In the coding region eSNVs, 5281 (43.6%) were non-synonymous eSNVs. There were 24,265 eSNVs present in 4 or more replicate cultures of hCMEC/D3 cell line. The mutation frequency for eSNVs is summarized at gene level. Considering the mutation rate is higher with longer genes, we normalized the gene level mutation frequency by gene length. Most highly mutated genes included Humanin Like genes *MTRNR2L2* and *MTRNR2L8* which are correlated with Alzheimer's disease. Other highly mutated genes are *CXCL11*, HLA family, *CITED4, PPP1R15A, ADRB2*, and *LDOC1L* as shown in Figure 1.

### Non-coding RNAs

#### CircRNAs

We identified 11 circRNAs in the BBB replicates, of which 5 circRNAs were found within the mitochondrial genome. Among the remaining 6 circRNAs, 5 were located on the chromosome 6, spanning HLA gene family, and another circRNA was on chromosome 11, overlapping gene *IFITM2*. Of the 11 circRNAs identified, two circRNAs were found in five or more samples, and six had only one sample supporting the evidence of that specific circRNA.

#### LincRNAs

We identified 13,218 lincRNAs in the replicates, of which 7256 are known (Gencode version 19) and 5962 are novel lincRNAs. Of the lincRNAs identified, 1620 known and 5370 novel lincRNAs were detected in all 8 BBB samples. Amongst the novel lincRNAs, 5865 contained single exons and 97 lincRNAs were multi-exon transcripts with 2 or more exons. We also observed that 2010 novel lincRNAs overlap protein-coding genes and the remaining 3952 lincRNAs were found neighboring one or more protein-coding genes. Information of distance of a lincRNA to its corresponding protein-coding gene (upstream indicated by a negative number and downstream indicated by a positive number) can be found in the BBBomics hub.

#### MicroRNAs

The miRNA-seq data was obtained for two replicate BBB monolayers. Employing our pipeline, we identified 2384 microRNA counts; of those identified only 578 microRNAs were expressed (with raw median microRNA count >20) in two replicates of hCMEC/D3 cell line. Both raw and normalized counts for microRNA along with target genes are listed in the BBBomics hub. Gene name or the microRNA name can be easily queried to obtain expression of the microRNA in BBB cell lines.

### Pathways

**Figure 2:**
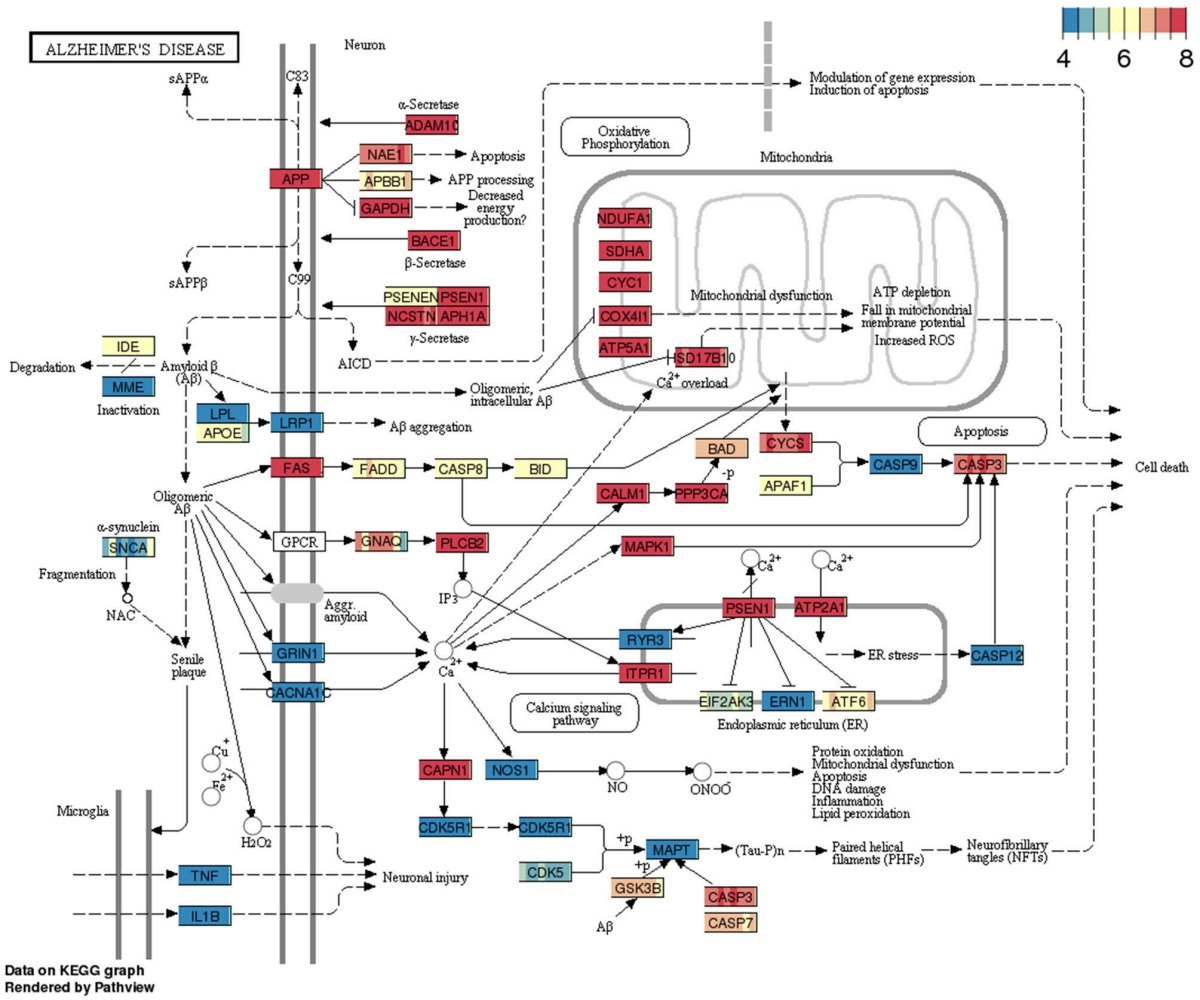
**Example pathway (Alzheimer's disease) overlaid with gene expression data from BBB cell line**. The pathway consists of a snapshot of genes expression profiles in replicate cultures of hCMEC/D3 cell lines.

The gene expression profiles from RNA-sequencing analysis that displayed a minimum of 5 annotated RNA genes were overlaid on KEGG pathways using pathview package. To show pathway data utility, we have queried Alzheimer's disease (AD) pathway from BBBomics hub (Figure 2). Each gene or node in the pathway is represented by eight bands; each band represents the normalized data counts of replicates. Pathway expression gradients for each replicate were depicted with respect to the 25th and 75th quantiles of the pathway expression matrix. It is widely believed that the BBB disruption is associated with the Alzheimer's pathology, and is also implicated in the impaired clearance of amyloid-β proteins. The hCMEC/D3 cell cultures would thereby represent homeostatic BBB, prior to the onset of Alzheimer's disease. In Figure 2 pathway diagram, the 25th quantile is ~4 CPM (as normalized be CQN) and the 75th quantiles is 8 CPM. Since the ranges translate to raw reads of 2048–32,768, and the CQN is shifted by ~7 [which is equivalent to 2^^(*CPM*+7)^], we may presume that the pathway is highly expressed in hCMEC/D3 cells. More details of pathway data interpretation is provided in Supplementary Methods. The pathway data are expected to provide insight into the expression levels of genes of interest and enable decision making on gene expression manipulations\functional studies of hCMEC/D3 cell lines.

## Discussion

In this work, we present RNA-seq and miRNA-seq data for the hCMEC/D3 BBB cell line, in an easily accessible database, with user-friendly interface fortified with effective querying tools. This web portal provides access to gene expression, alternate splice profiles, mutations (eSNVs), non-coding RNAs, and pathways that regulate BBB physiology. We have also made available, five additional microarray datasets for hCMEC/D3 cell line through our web portal. In future, we plan to treat hCMEC/D3 cell lines with a variety of drugs and integrate existing hCMEC/D3 microarray, RNA-Seq, and microRNA data with the perturbed omics data. Overall, this comprehensive BBBomics hub is expected to enable the researchers and computational biologists to navigate the underexplored frontier of the neurovascular unit.

## Author contributions

KRK and KKK were involved with design, data generation, data analysis, and manuscript preparation. KT, AN, XT were involved with design, data analysis, and manuscript preparation. MB and NJ were involved with handling of data and development of database. VL was involved with design and preparation of the manuscript. SS was involved with design, data generation, and manuscript preparation.

### Conflict of interest statement

KRK, KKK, KT, AN, XT, MB, NJ and SS declare that the research was conducted in the absence of any commercial or financial relationships that could be construed as a potential conflict of interest. VL serves is a consultant for Bayer Schering Pharma, Philips Molecular Imaging, Piramal Imaging and GE Healthcare and receives research support from GE Healthcare, Siemens Molecular Imaging, AVID Radiopharmaceuticals, the NIH (NIA, NCI), and the MN Partnership for Biotechnology and Medical Genomics. The reviewer CZ and handling Editor declared their shared affiliation, and the handling Editor states that the process nevertheless met the standards of a fair and objective review.
