# Supplementary material for: BBBomics-Human Blood Brain Barrier Transcriptomics Hub
Source: Front Neurosci. 2016 Mar 1;10:71. doi: 10.3389/fnins.2016.00071 (PMC4771746; doi:10.3389/fnins.2016.00071)
Supplement: Supplementary file 2 [file Presentation1.PDF]

Supplementary Material

BBBomics - Human Blood Brain Barrier Transcriptomics Hub

Krishna R. Kalari[1,*], Kevin J. Thompson [1], Asha A. Nair[1], Xiaojia Tang[1], Matthew A. Bockol[1], Navya Jhawar[1], Suresh K. Swaminathan[2], Val J. Lowe[3], Karunya K. Kandimalla[2,*]

*** Correspondence:**

Krishna R. Kalari Kalari.krishna@mayo.edu

Karunya K. Kandimalla kkandima@umn.edu

Supplementary Methods

Cell culture: The immortalized human cerebral microvascular endothelial cell line (hCMEC/D3) was kindly provided by P-O Couraud, Institut Cochin, France. The cells were cultured in EBM-2 endothelial basal media (Lonza, Walkersville, MD) containing 5% v/v fetal bovine serum (Atlanta biologicals, Flowery Branch, GA), 1% v/v penicillin-streptomycin (Corning, Walkersville, MD), 1.4 µM hydrocortisone (Sigma-Aldrich, St Louis, MO), 5µg/ml ascorbic acid (Sigma-Aldrich, St Louis, MO), 1% v/v chemically defined lipid concentrate (Life Technologies, Grand Island, NY), 10 mM HEPES, 1 ng/ml bFGF (Peprotech, Rocky Hill, NJ). The hCMEC/D3 cells were seeded on collagen-coated Transwell® filters and cultured) at 37 ° C under 5% CO_2 in a humidified chamber.

Illumina TruSeq v2 mRNA and microRNA Protocol: RNA libraries for eight replicates of polarized hCMEC/D3 cell monolayer were prepared according to the manufacturer's instructions using TruSeq RNA Sample Prep Kit v2 (Illumina, San Diego, CA). Briefly, poly-A mRNA was purified from total RNA using oligo dT magnetic beads. The purified mRNA was fragmented at 95°C for 8 min and eluted from the beads. Double stranded cDNA was made using SuperScript III reverse transcriptase, random primers (Invitrogen, Carlsbad, CA), and DNA polymerase I and RNase H. The cDNA ends were repaired and an "A" base is added to the 3' ends. TruSeq paired end index DNA adaptors (Illumina) with a single "T" base overhang at the 3' end were ligated and the resulting constructs were purified using AMPure SPRI beads from Agencourt. The adapter-modified DNA fragments were enriched by 12 cycles of PCR using Illumina TruSeq PCR primers. The concentration and size

distribution of the libraries were determined on an Agilent Bioanalyzer DNA 1000 chip and Qubit fluorometry (Invitrogen, Carlsbad, CA). The average RNA integrity number for 8 replicates is 8.3 (+/-0.7). MicroRNA libraries were prepared from 1 ug of total RNA according to manufacturer's instructions for the NEBNext Multiplex Small RNA Kit (New England Biolabs; Ipswich, MA). Libraries (3 samples per lane) were loaded onto paired end flow cells at concentrations of 8-10 pM to generate cluster densities of 700,000/mm^2 following Illumina's standard protocol using the Illumina cBot and cBot Paired end cluster kit version 3. The flow cells were sequenced as 51 X 2 paired end reads on an Illumina HiSeq 2000 using TruSeq SBS sequencing kit version 3 and HCS version 2.0.12.0 data collection software. Base-calling was performed using Illumina's RTA version 1.17.21.3.

User Manual: The user manual for web-portal is available at the following link

http://bioinformaticstools.mayo.edu/bbbomics/index.html

Using BBBomics Site

The web portal is divided into coding, noncoding, and pathway sections. Coding and noncoding RNAs are extracted from the RNA-Seq data, whereas the micro RNAs are obtained from microRNA-Seq data. The coding section constitutes of gene expression, alternate splicing, and SNVs; the noncoding section displays circular RNA and lincRNAs. Expressions of various genes involved in the KEGG Pathways could be searched using gene name (symbol), pathway ID, or pathway name.

Note that the query fields are pre-populated to provide a search string reference. Table queries are implemented as grep string matches and multiple queries can be run simultaneously. For instance a user can run an expression query: *FOXA1 FOXC1*.

Similarly, the pathways can be searched as *Alzheimer ECM* to yield both HSA05010 (Alzheimer's) and HSA04512 (ECM) pathway representations. In the Pathway query table we observe the 'bile secretion' is quoted and searched as single string, in the case of pathways utilizing apostrophe's in the pathway name, such as *Alzheimer's disease* would require the use of double quotes.

Gene Expression:

An individual gene expression could be searched by the gene symbol; if multiple genes are searched, they must be separated by a space. When a gene name is entered both raw and normalized gene expression counts are obtained for eight replicates. The raw gene expression table provides chromosome, gene name, expression counts, start and stop positions obtained using mapRseq workflow. Normalized data for the same gene is also displayed below the raw counts table. The raw gene counts were normalized by the CQN method that performs gene length correction; normalizes for total sequencing depth; and GC content with some offset parameters. Display of NA for any gene of interest in the normalized section means that the gene is not expressed, i.e., the median raw counts across the samples is less than 32.

Alternate Splicing:

If a gene of interest has multiple transcripts, both supporting read counts and Bayesian determined ratios (phi) counts will be displayed. The higher phi ratios (Bayesian probabilities) represent the more predominant isoforms. These counts were obtained by employing the MISO tool.

The MISO documentation nicely illustrates how the junction reads are interpreted to identify the exon skip event differentiating the two isoforms. For example, as shown below the read depths for exons 1 and 3 are both 4, and cannot be confidently assigned to either isoform. The read depth for the junction spanning evidence for the exon skip (0,1) is 3 and assigned to isoform 2. Conversely, supporting read evidence for the exon retention is also 3 and assigned to isoform 1. While there are 9 supporting reads, the 9 reads constitute a read depth of 3 covering the retained exon. The phi ratios of the two isoforms would then be 3:3 (0.5 for each). For further details please consult the MISO manuscript.

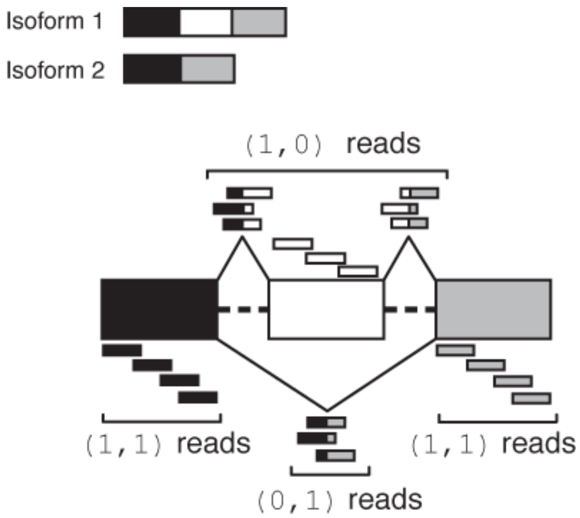

We have provided URL links to ENSEMBL gene models, which were found to have multiple isoforms in the replicated hCMEC/D3 sequencing data. Enabling the transcript view table, will provide relevant isoform information about the end user's gene of interest. Additionally, we have provided ENSEMBL transcript links with in the splicing query result. Please note that some identified transcript links may no longer be supported by the current ENSEMBL build version. The generated transcript ids are based upon the provided hg19.gff3 file, built in 2012.

Expressed Single Nucleotide Variants (SNV):

For a gene of interest, the expressed SNV search provides the variants that are present in the RNA sequencing data obtained from 8 replicates of hCMEC/D3 monolayers. When a gene is queried, the chromosome, chromosomal position, reference allele, and alternate allele found in the sample at that particular nucleotide position are displayed. A variant will also be given high, medium, or low confidence scores, based on its presence in multiple samples.

Functional annotation of the SNV (exonic, 5' UTR, 3' UTR):

Exonic annotations will be further displayed as synonymous or non-synonymous. Amino acid changes in the translated protein will be provided for each variant. Information on the conservation among species and segmental duplication annotations are obtained from the UCSC genome browser. The SNV variant in DBSNP, thousand genome, or ESP6500 (exome variant server) databases is reported. This will help us differentiate reported variants from the novel variants that may be specific to the hCMEC/D3 cell line. The variant is also classified as deleterious using AVsift, which reports predicted impact of the protein.

MicroRNA:

MicroRNA could be searched with matured microRNA name or gene name. If a gene name is entered, any microRNA that can bind to the gene will be listed. Moreover, the number genes that microRNA could target will also be listed. In addition, normalized data is also represented. Raw reads were normalized to a million and were further computed by dividing each micro RNA raw read count by the total number of micro RNA reads to arrive at the normalized reads for each sample.

CircularRNA:

There were only 11 circular RNAs found. Hence, the complete table with the raw counts was provided without a search option.

LincRNA (Long non-coding RNAs):

LncRNAs are obtained for all the eight replicates along with the annotation to the closet gene name and distance. The length and start/stop information of a particular LncRNA could be searched in the context of any gene of interest by providing the gene name. The normalized and raw counts are displayed. The raw values for each lincRNA were normalized to a million and corrected for the lincRNA length to obtain the normalized reads (CPM).

KEGG Pathways:

Pathways could be searched with constituent gene names (symbol), pathway ID, or pathway name. With the gene name as the search term, all the associated pathways could be retrieved. The retrieved information comprises of KEGG pathway outline on which the gene expression is overlaid. Blue colored boxes represent low gene expression; whereas the red boxes represent high expression. Moreover, the pathway search could be performed by KEGG pathway ID as well as by the KEGG related to insulin signaling/trafficking will be displayed.

There are several components to the pathview representations of the KEGG pathways, which need to be understood. First we've constructed each pathway expression matrix, with *n* genes in the pathway by the 8 replicated hCMEC/D3 cultures. For HSA05010 (Alzheimer's Disease), that expression matrix comprises 168 genes. We observed little biological variation among the replicates represented by their gene-nodes, since most of the gene-nodes appear as one solid color.

In addition, we observed that there are not 168 nodes representing all the genes. Nodes such as CALM1 represent 6 transcriptional variations of calmodulin (6 gene models, not isoforms). Of several options the Pathview provides to represent multi-gene nodes, we have chosen to use the mean, which isalso the default option in the software. In the example of CALM1, the node is represented for each sample by the mean of the six genes.

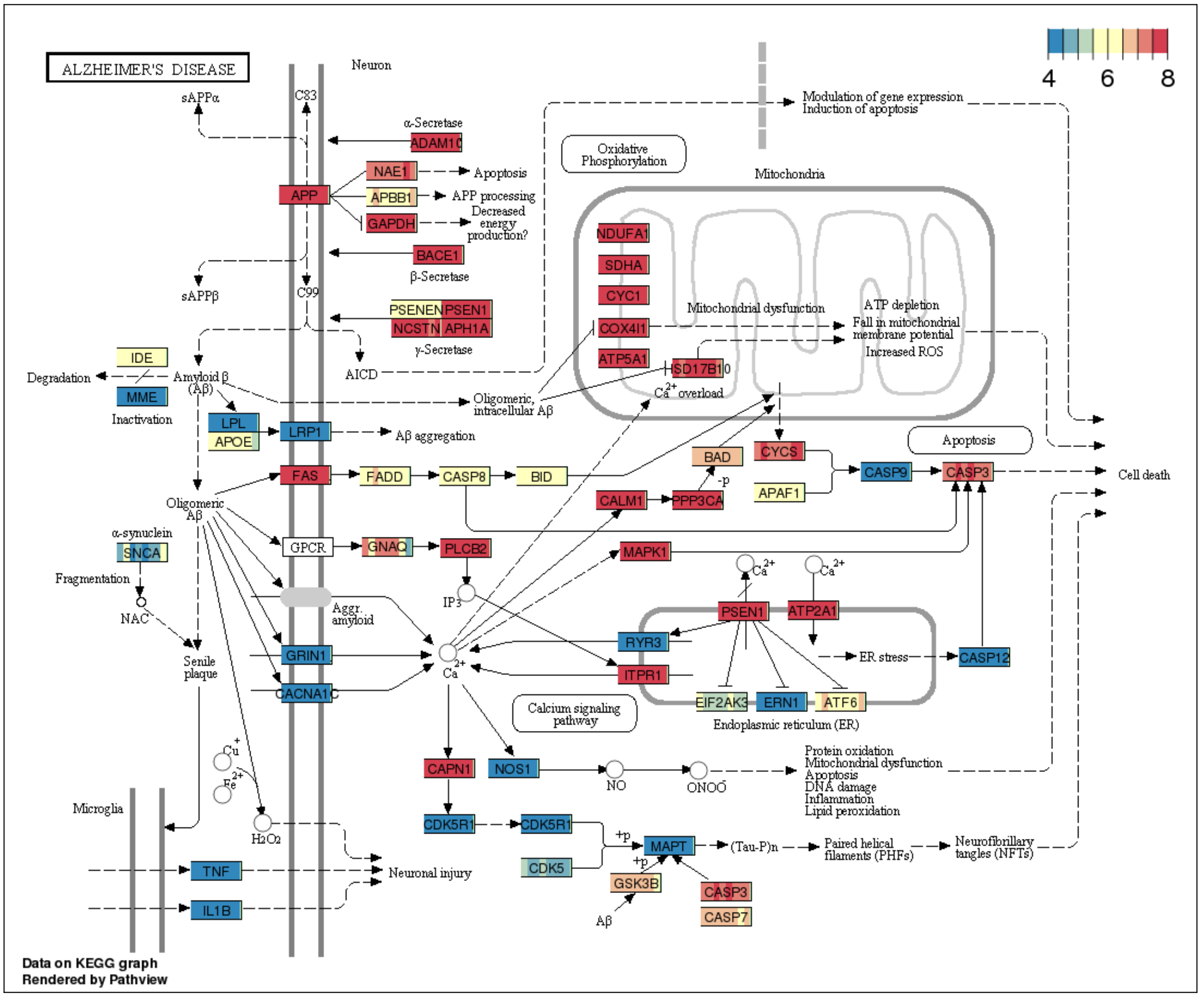

It is widely believed that the blood brain barrier disruption is associated with the Alzheimer's pathology, and is also implicated in the impaired clearance of amyloid-β proteins. The hCMEC/D3 cell cultures would thereby represent homeostatic blood brain barrier, prior to the onset of Alzheimer's disease. In this pathway diagram, the 25th quantile is approximately 4 CPM (as normalized be CQN) and the 75th quantiles is 8 CPM; hence, we can presume that the pathway is highly expressed in hCMEC/D3 cells, since the ranges translate to raw reads of 2,048 to 32,768, and the CQN is shifted by approximately 7; 2^ $^{CPM+7}$). We observed that receptors such as (LRP1, GRIN1, and CACNA1C), shaded in blue, are all down regulated with respect to the rest of the pathway. Similarly, we see that the caspase 9 and 12 (as well as caspase 8), which are associated with apoptosis are also down regulated. In addition, IDE and MME, which are implicated in the degradation of amyloid-β proteins are down-regulated.. In contrast, if we examine the pathway describing malaria pathogenesis, which does not primarily involve the blood-brain barrier,

particularly in the cell lines,we observe that raw read counts range from 32 to 4,096. The quantiles of this pathway expression matrix is skewed and the higher expression levels are being driven by some rather ubiquitous genes (TGFB1, VCAM11, PECAM1, ICAM1, MET, etc.). The expression does not support the relevance of the blood-brain barrier in this pathway.

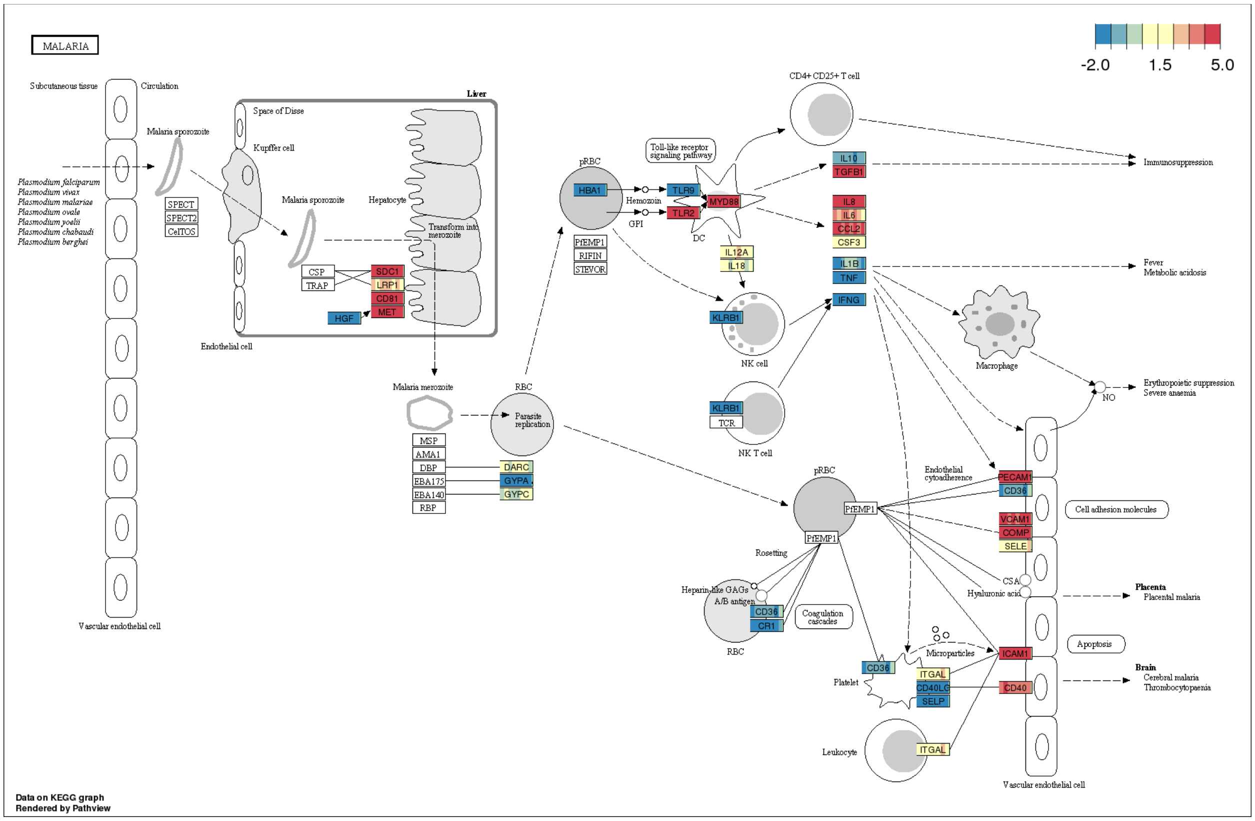

Finally, in pathview representations, the default representation normalizes each pathway (-1,1). We have chosen to represent the quantile, in order to facilitate the comparison among pathways, as we've illustrated above for malaria and Alzheimer's disease. Normalized pathways could hinder the ability to contrast between pathways. Conversely, if the contrast gradient represented the 25th (-2) and 75th (6) quantiles of the entire expression matrix (23,346 genes), the expression differences in the relevant pathway (such as Alzheimer's disease) would be lost (the entire pathway would be red).

Finally, limitation of the pathview package is the inability to label the color key legend. The low and high limits are provided to the pathview package and represented as the extremes on the color key

legend. The middle value of the color key legend does not necessarily represent the median (50th quantile) value of the pathway expression matrix. Rather, it is simply the mid-point between the two color key extremes. Rastering the image with our own color legend is a possibility, which will be explored in the future analyses posted on the BBBomic website

Data download: All data generated for this publication is now available through BBBomics web link. In addition to our data we have also made the following data available through our download link

1. GSE44694 miRNA array, mRNA microarray PMID: 24604078

ftp://ftp.ncbi.nlm.nih.gov/geo/series/GSE44nnn/GSE44694/matrix/GSE44694-GPL14767_series_matrix.txt.gz

ftp://ftp.ncbi.nlm.nih.gov/geo/series/GSE44nnn/GSE44694/matrix/GSE44694-GPL6883_series_matrix.txt.gz

(MiR-155 promotes blood-brain barrier dysfunction in neuroinflammation

http://www.ncbi.nlm.nih.gov/geo/query/acc.cgi?acc=GSE44694)

2. GSE29932 mRNA microarray

ftp://ftp.ncbi.nlm.nih.gov/geo/series/GSE29nnn/GSE29932/matrix/GSE29932_series_matrix.txt.gz

(To identify specific glycosylated structures on brain endothelial cells that mediate rolling, adhesion, and diapedesis of leukocytes in the brain

http://www.ncbi.nlm.nih.gov/geo/query/acc.cgi?acc=GSE29932)

3. GSE44692 mRNA microarray PMID: 24604078

ftp://ftp.ncbi.nlm.nih.gov/geo/series/GSE44nnn/GSE44692/matrix/GSE44692_series_matrix.txt.gz

MiR-155 promotes blood-brain barrier dysfunction in neuroinflammation (part 1 of #1 above)

http://www.ncbi.nlm.nih.gov/geo/query/acc.cgi?acc=GSE44692

4. GSE44693 mRNA microarray PMID: 24604078

ftp://ftp.ncbi.nlm.nih.gov/geo/series/GSE44nnn/GSE44693/matrix/GSE44693_series_matrix.txt.gz

MiR-155 promotes blood-brain barrier dysfunction in neuroinflammation (part 2 of #1 above)

http://www.ncbi.nlm.nih.gov/geo/query/acc.cgi?acc=GSE44693

5. GSE45880 mRNA microarray pubmed id: 24050303

ftp://ftp.ncbi.nlm.nih.gov/geo/series/GSE45nnn/GSE45880/matrix/GSE45880_series_matrix.txt.gz

Cytokine-induced changes in the gene expression profile of human cerebral microvascular endothelial cell-line, hCMEC/D3

http://www.ncbi.nlm.nih.gov/geo/query/acc.cgi?acc=GSE45880
